# A 9,000 Year History of Seal Hunting on Lake Baikal, Siberia: The Zooarchaeology of Sagan-Zaba II

**DOI:** 10.1371/journal.pone.0128314

**Published:** 2015-05-26

**Authors:** Tatiana Nomokonova, Robert J. Losey, Ol’ga I. Goriunova, Alexei G. Novikov, Andrzej W. Weber

**Affiliations:** 1 Anthropology Program, Community, Culture and Global Studies, The University of British Columbia—Okanagan, Kelowna, British Columbia, Canada; 2 Department of Anthropology, University of Alberta, Edmonton, Alberta, Canada; 3 Laboratory of Archaeology and Paleoecology, Institute of Archaeology and Ethnography, Siberian Branch of Russian Academy of Science, Irkutsk, Russian Federation; ICREA at the Universitat Autònoma de Barcelona, SPAIN

## Abstract

Sagan-Zaba II, a habitation site on the shore of Siberia’s Lake Baikal, contains a record of seal hunting that spans much of the Holocene, making it one of the longest histories of seal use in North Asia. Zooarchaeological analyses of the 16,000 Baikal seal remains from this well-dated site clearly show that sealing began here at least 9000 calendar years ago. The use of these animals at Sagan-Zaba appears to have peaked in the Middle Holocene, when foragers used the site as a spring hunting and processing location for yearling and juvenile seals taken on the lake ice. After 4800 years ago, seal use declined at the site, while the relative importance of ungulate hunting and fishing increased. Pastoralists began occupying Sagan-Zaba at some point during the Late Holocene, and these groups too utilized the lake’s seals. Domesticated animals are increasingly common after about 2000 years ago, a pattern seen elsewhere in the region, but spring and some summer hunting of seals was still occurring. This use of seals by prehistoric herders mirrors patterns of seal use among the region’s historic and modern groups. Overall, the data presented in the paper demonstrate that Lake Baikal witnessed thousands of years of human use of aquatic resources.

## Introduction

People and seals have formed long-lasting relationships in many parts of the world. This is especially true in the north, where these animals are a major part of societies’ economies and overall well-being, both presently and in the distant past [1–14]. Understanding the histories of these relationships can be informative about a wide range of long-term processes, from the evolution of subsistence economies, through human impacts on aquatic ecosystems, to the variable ways of using and experiencing a landscape and its resources. Archaeological evidence of these histories in the maritime north often extends only a few thousand years into the past, in part as a result of the erosion of coastal sites due to Holocene sea level rise, but also because of poor faunal preservation in some settings. With the exception of a few sites on the Russian Pacific coast [15–17], the long-term archaeological history of sealing in Arctic and Subarctic Asia is rather poorly known.

**Fig 1:**
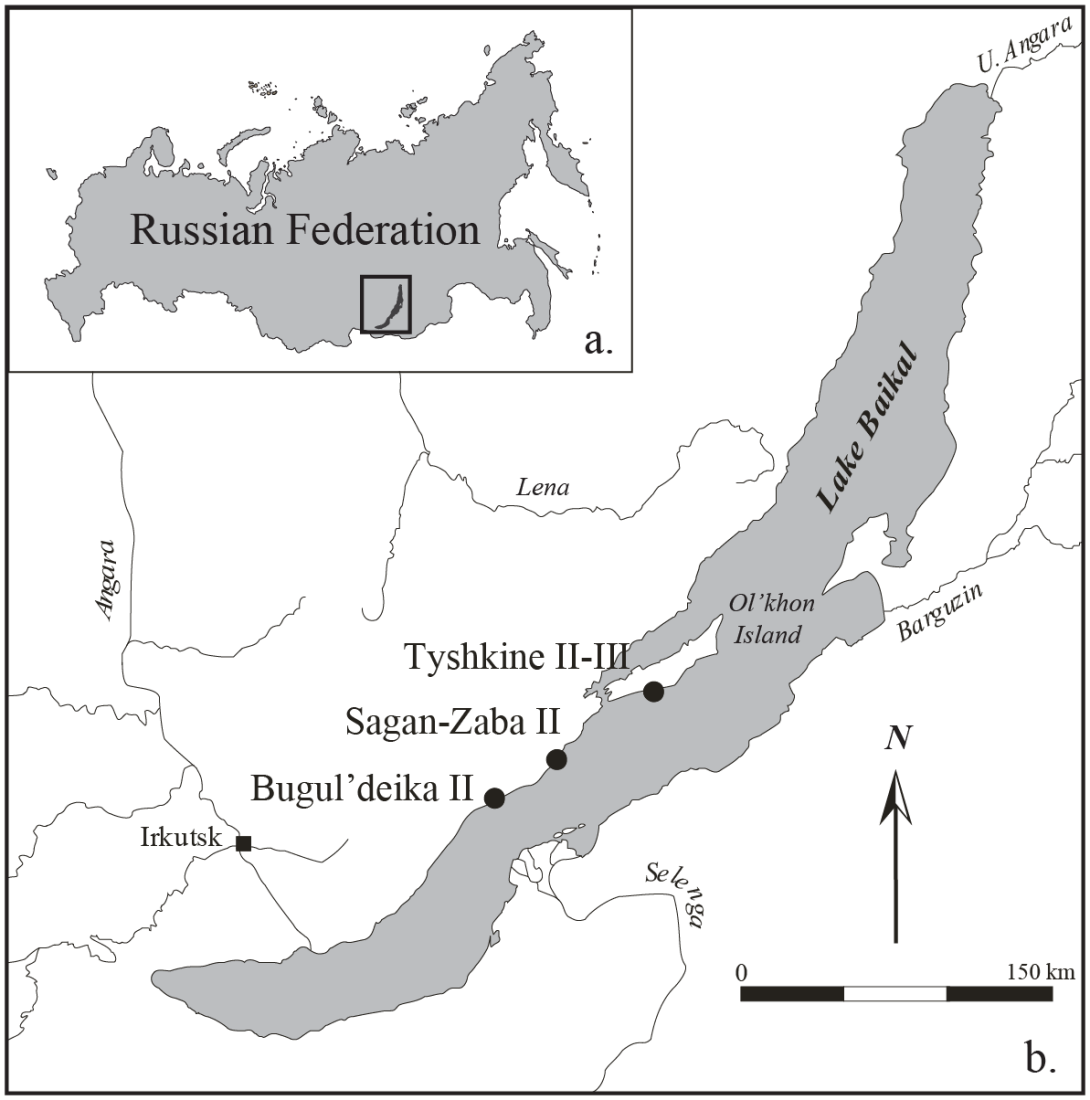
Study area: a. Lake Baikal, Russian Federation; b. archaeological sites mentioned in the text.

Lake Baikal, Siberia (Russian Federation; Fig 1), is a compelling and unique place to study long-term interactions between people and seals for several reasons. First, this massive freshwater lake in the interior of Asia has been home to an endemic seal species, the *nerpa* or Baikal seal (*Phoca* or *Pusa sibirica*), for several hundred thousand years [18], spanning the entire period over which humans have occupied this portion of North Asia. Second, the archaeological record of the Lake Baikal shoreline is relatively rich, especially for the Holocene, and includes ample evidence for the use of these animals. Much of this record, particularly from habitation sites, remains poorly documented, but it is clear that by at least the Middle Holocene, seals were regularly being used by the region’s foragers [19–24]. Third, archaeological evidence clearly shows that in this region sealing also was carried out by pastoralists, who first migrated to this broader region around ~3500 cal. BP [25–27]. In the more recent historic period, Evenk, Buriat, and Russian settlers sealed on Lake Baikal too, especially from the 17^th^ through the 20^th^ century, when seal products were used in various regional industries [28–32]. Further, hunting for Baikal seals continues to be a living tradition among some local people, particularly the Buriat [33]. In sum, Lake Baikal appears to be home a very long record of human interaction with pinnipeds, and this interaction involves a diverse suite of past and modern groups.

The Lake Baikal region is a major area of emerging archaeological scholarship on northern hunter-gatherers [23, 34–36]. Zooarchaeological data on human subsistence practices, however, have remained rare until quite recently [20, 27, 37–40], with most research on Holocene subsistence practices and diets in this region largely relying on the geochemical analysis of human skeletal remains [19, 23–24, 41–43]. This paper is a major step towards filling this lacuna. Until quite recently [39, 40, 44], the ancient history of seal use on Lake Baikal had been explored solely through the study of thin sections of archaeological seal canines, which were examined for age and season of death [22, 45]. This research suggested that seal use at Lake Baikal focused on the early spring period when the seals basked on the lake ice, that sealing was most intensive during the Bronze Age (~5000 to 3400 cal. BP) and that in some mortuary contexts yearlings were preferentially used over the other age groups. However, the rest of the faunal assemblages from which the canines were obtained remained otherwise unstudied. As a result, it was impossible to assess the relative importance of seals at these sites, nor was it clear how representative the canines were of the overall seal assemblages. Furthermore, the vast majority of the analyzed canines were from poorly documented contexts, most of which were only typologically dated, and none of the sites from which the teeth were excavated were sieved, almost certainly resulting in faunal assemblages biased towards the remains from large fauna.

To address these shortcomings, the Baikal Archaeology Project (centered at University of Alberta, Canada) carried out excavations from 2006–2008 at the Sagan-Zaba II habitation site on the western shore of the lake (Fig 1). All necessary permits were obtained for the described study, which complied with all relevant regulations. This site was the first in the Lake Baikal region to be systematically sieved, extensively AMS radiocarbon dated, and fully analyzed using contemporary zooarchaeological methods [40, 46–48]. These excavations produced ~74,000 animal skeletal remains dating to the Holocene, including just over 16,000 Baikal seal specimens, which constitute the majority of the identified faunal remains at the site. Sagan-Zaba II thus provides the best currently available dataset with which long term human interactions with Baikal seals can be explored, and perhaps the longest and most extensive record of seal use from a single location within North Asia.

## Background and Setting

Foraging groups were first present in Lake Baikal region in the Late Pleistocene, perhaps starting around 70,000 years ago [49, 50]. The shores of lake, however, have yielded few possible Pleistocene sites, and these consist largely of surface materials. Changes in lake level during the Holocene may have stranded or eroded such old sites, but the history of these changes is no currently well documented. Numerous Holocene archaeological sites have been found around the lake, with many of the earliest ones located along its southwest shoreline. The most extensively studied are those formed by Middle Holocene foragers (general Holocene subdivisions follow [51]) who in some locations created formal cemeteries and made extensive use of the lake’s aquatic fauna, including its seals [19–21, 24, 35, 41–43, 52–53]. The Middle Holocene mortuary record is marked by a ~1100 year period (~6800 to 5700 modeled cal. BP) of absence of human burials, and human populations before and after this hiatus appear to be genetically discontinuous [34, 54]. During the Late Holocene, substantial changes occurred in this region with the sequential arrival of at least four Eurasian pastoralist populations, most commonly thought to be related to groups known more broadly as Scythians, Xiong-nu, Turks, and Early Mongols [55]. Their occupation of the Baikal shoreline left behind numerous archaeological sites, including habitation sites, fortifications, rock art, cemeteries, and stone features associated with animal sacrifices [25–27, 56].

One of the key reasons for the density of human settlement in the Baikal region compared to adjacent areas of Siberia is that the lake and its nearby rivers offer an abundance of aquatic food resources [57], including several endemic species, with perhaps the most well known being the Baikal seal. This freshwater seal is only found in Lake Baikal and portions of its tributaries. It shares lifecycle and behavioral patterns with other small northern ice-adapted seals, and is genetically and morphologically most closely related to the ringed seal (*Pusa hispida*; [30, 58–59]). The *nerpa* can grow up to 1.8 m long and weigh as much as 130 kg, with the males tending to be slightly larger than the females [30]. Pups are born with white fur, which changes within a two-month period to a silver-grey color, and this color is then retained throughout adulthood. Historically, the seal population has been estimated to number as many as 104,000 individuals [18].

For Baikal seals, the ice regime is a crucial ecological factor, as it affects many aspects of their life history and behavior. Lake Baikal freezes over completely from January to mid-May, and seals during this period distribute themselves unevenly across the lake. Just prior to freeze-over, females concentrate mainly in the deep open waters. When these regions of the lake are finally ice-covered in January, the females form dens within the ice and snow that are used as birthing areas for their pups. Adult males and juveniles try to extend their open-water feeding period as much as possible just before ice formation, and concentrate in areas that are ice-free. After the lake is ice-covered, the adult males are mostly found on the ice over the deeper portions of the lake, while the juveniles are more common nearer the shoreline. During the ice-cover period, seals separate from each other, with the spacing between their breathing holes and dens typically being greater than 100 m. Birthing usually starts at the end of February and lasts through the beginning of April. Lactation lasts approximately 2.5 months, partially overlapping with the mating period [30]. Female seals reach sexual maturity between the ages of two to five years, and the males between five and eight years of age. Mating occurs in the water, mainly in the first half of April, with pregnancy lasting 11 months.

With rising temperatures in April, breathing holes increase in size and leads (fissures) in the ice become more prevalent. At this time, seals lose up to 30% of their weight, as they spend significant time basking on the ice and moulting. During the ice break-up period in spring, from the second half of May until the end of June, the animals continue moulting and form aggregations of different densities on the remnant floating ice patches. In the summer, the animals can be found everywhere in the deep parts of the lake, and generally avoid its shallower sections. They often form aggregations on isolated rocky portions of the shoreline and on islands, sometimes consisting of up to a few thousand individuals. In the fall, the seals start to migrate to the north and northeast part of the lake to take advantage of the early forming ice, which forms a safer resting platform than the shoreline. The seals also sometimes form large groups on this first thin ice, or when resorting to using the shoreline as they wait for the ice to form. By January, they disperse into the deep-water areas of Lake Baikal, preparing for winter [30].

Remains of Baikal seals are often found among the faunal specimens recovered from habitation sites along the west and north shores of the lake, as well as at a few sites along the eastern shore. Some also have been found within the graves of Middle Holocene foragers, and in animal sacrifice features created by the region’s Late Holocene pastoralists [59]. However, large quantities of seal remains have only been found in three camp site locations, all along the western open shoreline proximate to the lake’s deepest waters, which are the seal’s preferred feeding and birthing habitats. These three sites are Bugul’deika II, Tyskhine II-III, and Sagan-Zaba II (Fig 1; [38–40, 44, 60]). Among these sites, Sagan-Zaba II has produced by far the largest seal assemblage.

## Sagan-Zaba II

**Fig 2:**
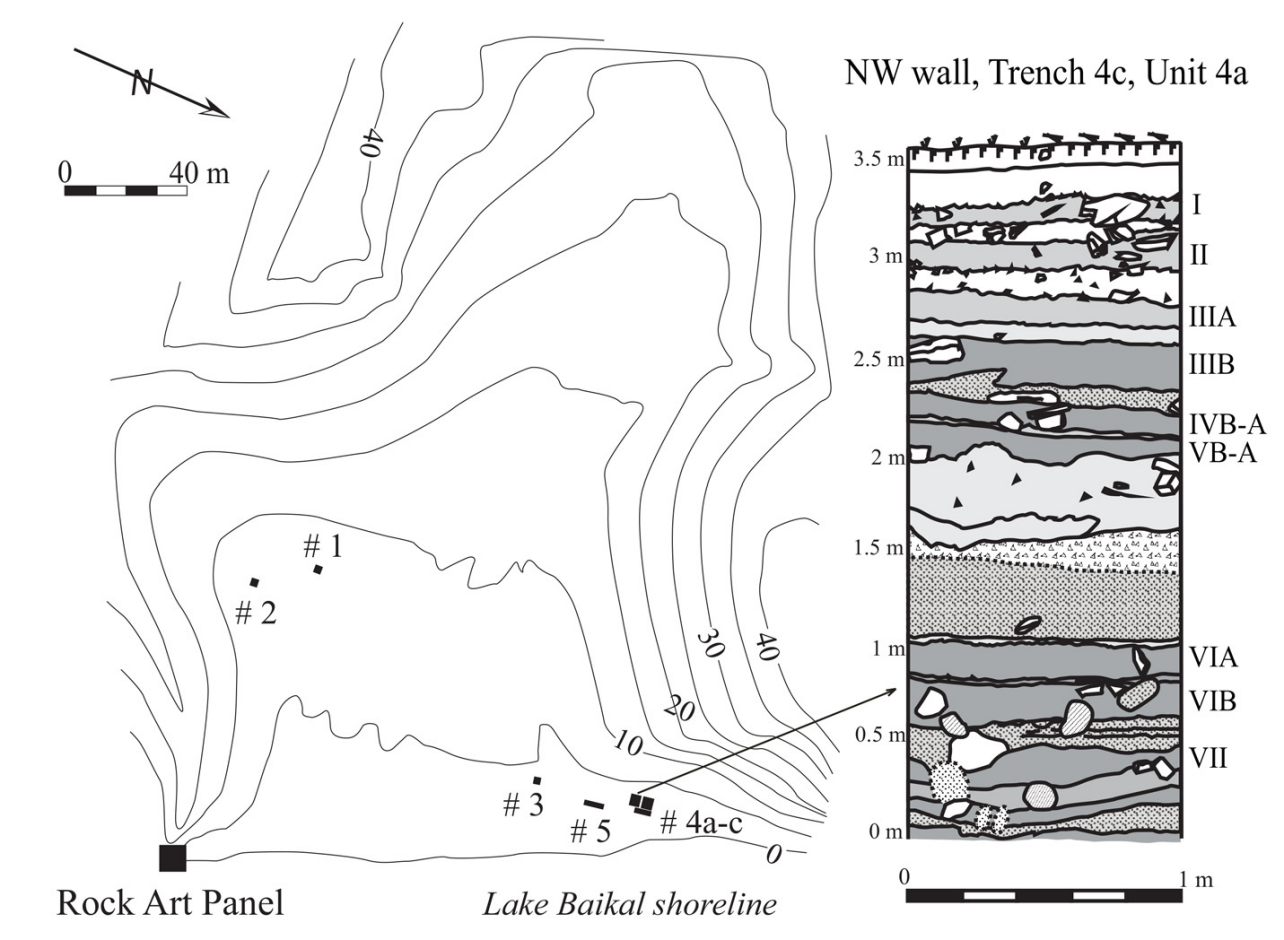
Map of Sagan-Zaba cove with locations of rock art panel and trenches (#1–3, 4a-c, and 5) indicated, and a stratigraphic column of trench 4c. Detailed description of site stratigraphy is provided in Nomokonova (2011) and Nomokonova et al. (2013b).

**Fig 3:**
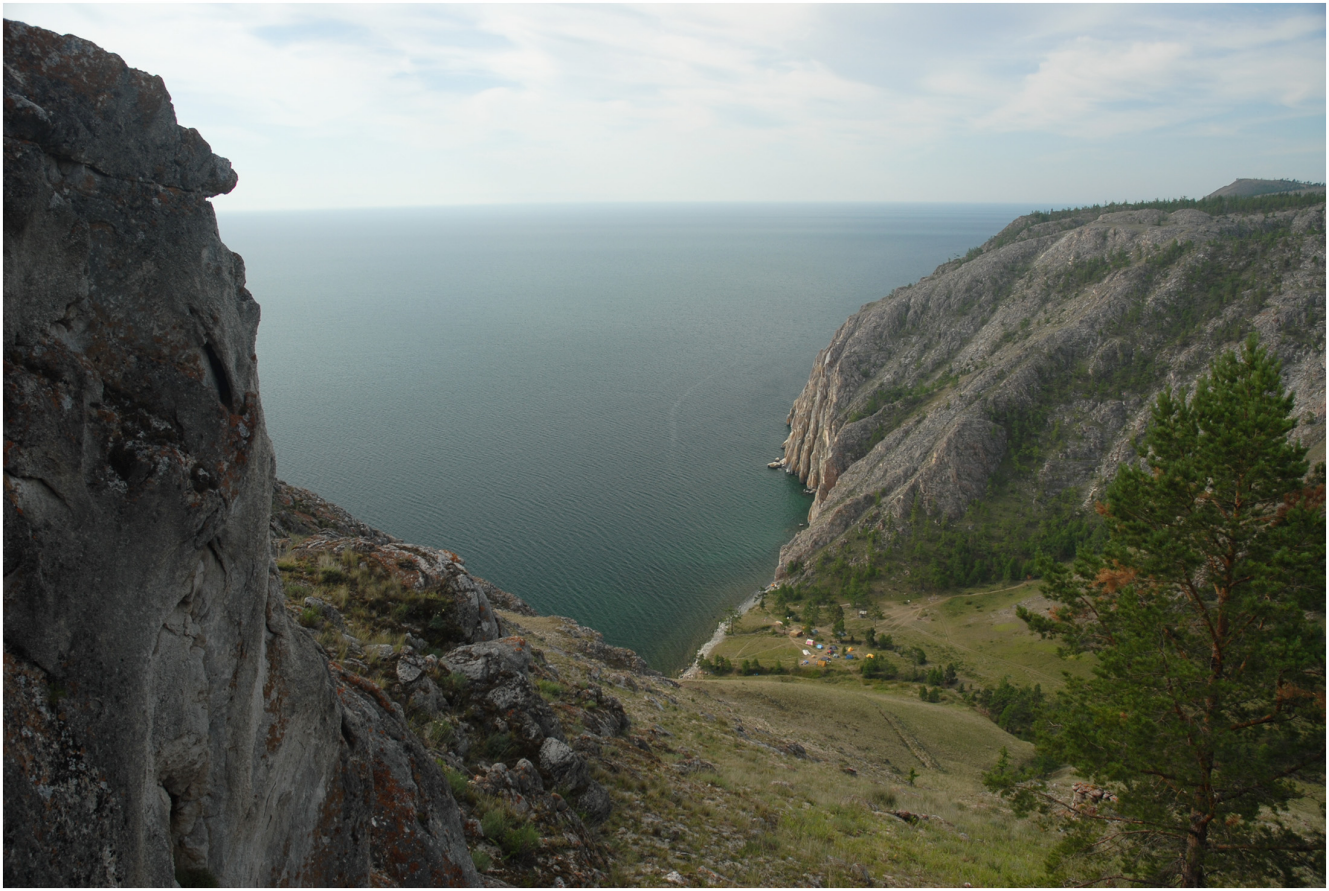
Photograph of Sagan-Zaba from the hill to the north of the cove, with the field camp tents visible on the valley floor. Photograph by A. Weber.

Sagan-Zaba cove is a ~200 m long funnel-shaped valley framed by steep hills, with a 5 m wide beach of cobbles and pebbles, and a 150 m wide terrace eroded by several small channels (Figs 2 and 3). The terrace holds the stratified archaeological materials described here. The rock cliffs along the shoreline are partially formed by white marble, leading to this place being named Sagan-Zaba, or “white bowl,” in the local Buriat dialect. The vertical cliff at the southwest end of the cove holds a large panel of rock art (Sagan-Zaba I) with images of humans, deer, birds, and riders on horseback. Based on their styles and content, these images are thought to have been made from ~4400 to 1000 cal. BP [61–62].

Test excavations at Sagan-Zaba revealed that some archaeological materials were present throughout the sediments in the floor of the valley, but were most well stratified and best preserved in a small area along the northeast shoreline. Our research thus centers on materials recovered from two trenches at Sagan-Zaba, numbered 4b and 4c (Fig 2), excavated in 2007–2008 in the northeastern portion of the cove, with the total volume of sediment sampled being 67 m^3^ [27, 40, 47, 63–64]. The Field Investigations Branch of the Institute of Archaeology, Russian Academy of Science provided excavation permits (# 286 and 325) for these excavations. The collection produced by these excavations is currently curated at the Irkutsk Laboratory of Archaeology and Paleoecology Institute Archaeology and Ethnography Siberian Branch of Russian Academy of Science, K. Marks Str., 1, Irkutsk, Russian Federation, 664003. There is no accession number for the collection, which is curated by site name. Note that five other exploratory trenches were also excavated across the terrace, but these produced only a smattering of archaeological materials and were not well-stratified; therefore, they are not discussed further here. Trenches 4b and 4c were excavated using trowels, all sediment was sieved over 3 mm mesh screens, and all objects found in situ were plotted in three dimensional position.

The sediments in trenches 4b and 4c contained 20 lithological strata primarily formed of talus, colluvium, and proluvial sediments [40, 48]. While the two trenches were separated by only a 1 m wide bulk of unexcavated sediments, the structure and genesis of the deposits in each trench are somewhat different. Specifically, trench 4c generally had more clearly distinguishable strata with less evidence of post-depositional disturbance, while the upper portion of trench 4b was more disturbed, containing several rodent burrows and in-filled cracks, the latter perhaps produced through seismic activity. This burrowing and cracking almost certainly resulted in some mixing of archaeological materials between strata [48]. Artifacts and faunal remains were found within seven lithological layers (Fig 2), each of which was assigned a roman numeral (VII–I), with further sublayers marked with a letter following the numeral (e.g., IIIB and IIIA). Overall, these layers produced just over 5600 artifacts and 74,000 faunal remains from occupations spanning the Siberian Mesolithic through ethnohistoric periods (see [21, 23, 27, 50] for culture history descriptions).

Chronological assessment of the archaeological materials recovered from the two trenches is based on 89 AMS radiocarbon dates on bones of ungulates and Baikal seals, all generated by the Oxford Radiocarbon Accelerator Unit. Analysis of radiocarbon dates and chronological evaluation of the site are provided elsewhere [47]. Here we use calibrated age spans for the layers based only on the ungulate bone dates (Table 1) as the seal bone dates all are affected by an old carbon offset of approximately 700 years [47]. Ungulate and seal bone dates are utilized to calculate estimates of the elapsed time intervals (ETIs) for sets of dates from individual layers (Table 1); both sets of date interval estimates are presented to demonstrate the general consistency in results from the two sets of dated faunal remains. The time interval estimates were generated using the Bayesian Radiocarbon Calibration Tool (BCAL) [47, 65]. The ETIs presented in Table 1 are used to compare the time spans for individual layers, and to estimate the deposition rates of faunal remains within each layer.

**Table 1:** Sagan-Zaba II chronology following [47].

Layer	Age span, calibrated years BP, 2 sigma	ETI for ungulate dates in years, 68% confidence interval	ETI for seal dates in years BP, 68% confidence interval
VII	9020–8650	1–257	1–244
VIB-A	8160–7880	1–79	1–222
VB-A	6750–6310	243–368	185–309
IVB-A	5590–4870	710–924	798–1023
IIIB	4440–2000	2174–2331	3052–5169
IIIA	1970–1540	264–998	n/a
II-I	1230–940	55–145	n/a

ETI is estimated time interval calculated (where possible) from multiple radiocarbon dates from each layer.

The AMS radiocarbon dating of Sagan-Zaba II (based on the ungulate dates) indicates that the cove was first occupied starting around 9020 cal. BP, followed by a series of episodes of site use spanning much of the rest of the Holocene. Faunal remains from the earliest periods of site use, namely layers VII and VI, date from 9020 to 8650 and 8160 to 7880 cal. BP, respectively. Each of these layers individually appears to represent less than 250 years of site use, based on the calculated ETIs, which are shown at the 68% confidence interval in Table 1. The Middle Holocene layers, namely VB-A and IVB-A, span from 6750 to 6310 and 5590 to 4870 cal. BP, respectively. These occupations are less temporally discrete, as the ETIs indicate uses of the cove spanning anywhere from several centuries to as much as one millennium (Table 1).

Chronology of the materials found within sublayer IIIB represent a complicated case. The dates from the layer range from 4400 to 2000 cal. BP, and the layer’s faunal remains and artifacts indicate that this layer contained materials deposited by both Middle Holocene foragers and later pastoralist groups [40]. Based on the current evidence, it is unclear whether both groups co-existed at the cove, alternated their use of the cove, or sequentially used this space. However, the radiocarbon dates suggest that faunal remains derived from both groups’ use of the cove represent two sets of fairly short occupations, which occurred around 4440–4250 and 2300–2000 cal. BP, respectively. Alternatively, the data can be taken to indicate that the cove was only intermittently used throughout the 2000 year time range represented by the dates (Table 1), with the materials from these occupation episodes becoming intermixed by post-depositional processes. Dates for layer IIIA range from 1970 to 1540 cal. BP with the ETI spanning several centuries. In other words, sublayer IIIB is of uncertain chronology and its faunal remains must be interpreted cautiously. The uppermost two layers, II and I, represent occupation of the cove from 1230 to at least 940 cal. BP and cannot be chronologically separated, as the radiocarbon dates from them substantially overlap one another (see [47] for details). The ETI value for the dates from these two layers combined indicate a relatively short period of deposition, not longer than 150 years (Table 1). Note, however, that there is also some typological evidence for use of the cove postdating this period, perhaps some time after 550 cal. BP [47].

## Seal Remains at Sagan-Zaba II

Faunal remains from Sagan-Zaba II were identified using an extensive comparative collection assembled by T. Nomokonova and R. Losey and curated in Irkutsk, Russian Federation. The bulk of the 74,000 specimens recovered from the site consisted of mammal bone fragments that could not be more specifically identified. The identified specimens numbered 19,669, and 82.8% of these (16,283 specimens) were from Baikal seals (Table 2). Given their dominance in the assemblage, and the very gracile nature of their skeletons, the majority of the fragments in the unidentified mammal category likely also are from Baikal seals.

**Table 2:** Faunal remains from Sagan-Zaba II trenches 4b-c.

Taxa	Common Name	VII	VIB-A	VB-A	IVB-A	IIIB	IIIA	II-I	NISP per Taxon
**Molluska**	**Mollusks**		6	61	2	4	1		74
**Pisces-unidentified**	**Fish**	107	1	14	52	141	107	92	514
Salmonidae	Whitefish, grayling			29		363	428	31	851
*Coregonus* sp.	Whitefish/omul'				3	8	6		17
*Thymallus articus*	Grayling	3				1	2	1	7
Cyprinidae	Carps				1	1			2
*Leuciscus baicalensis*	Siberian dace					1	1		2
*Rutilus rutilus lacus*.	Siberian roach						3		3
*Acipenser baerii baic*.	Baikal sturgeon		1	10	1	28	5	3	48
*Esox lucius*	Northern pike			3	11			1	15
*Lota lota*	Burbot				2				2
*Perca fluviatilis*	Eurasian perch			31	17	5	2		55
**Aves-unid.**	**Birds**	3		6	3	6	3	1	22
Anatidae	Ducks, geese, swan	2							2
*Anas* spp.	Ducks					1			1
Accipitrinae	Hawks, eagles				1				1
*Haliaeetus* sp.	Fish eagles	1	1						2
*Buteo* cf. *buteo*	Common buzzard					1			1
*Phalacrocorax carbo*	Great cormorant	9							9
*Corvus* cf. *corax*	Common raven		1						1
Emberizidae	Buntings, sparrows						1		1
**Mammalia-unid.**	**Mammal**	772	929	10105	19344	6767	7225	9018	54,160
*Equus* spp.	Horse					3	25	35	63
Artiodactyla	Even-toed ungulates	12	10	99	21	39	97	155	433
Cervidae	Deer	12	22	105	42	6	6	12	205
*Cervus elaphus*	Red deer	19	13	11	11	7	10	10	81
*Alces alces*	Elk		1	2	1		1		5
*Capreolus pygargus*	Roe deer	9	10	80	14	16	26	65	220
*Sus scrofa*	Boar	2	2	1	10				15
*Bos* spp.	Oxen, true cattle				2	12	47	110	171
Caprinae	Goat, sheep				1	22	58	86	167
*Ovis aries*	Sheep					5	5	7	17
*Capra hircus*	Goat					1		4	5
Carnivora	Flesh eaters	1	1	50	5	1		1	59
*Canis* spp.	Wolf/dog			2	1				3
*Vulpes vulpes*	Red fox			19				1	20
*Lutra lutra*	Otter					1			1
*Martes zibellina*	Sable						1		1
*Phoca sibirica*	Baikal seal	101	296	6644	8540	416	112	174	16,283
Rodentia	Rodents			4	17	31	37	14	103
*Lepus timidus*	Mountain hare			2	1	3	2		8
*Castor fiber*	Beaver			1		1			2
*Marmota* sp.	Marmot							1	1
Muridae	Mice, rats			1	6	27	18	3	55
*Urocitellus undulatus*	Eurasian ground squirrel			3	44	54	14	6	121
**Unidentified**	**Unidentified**	4		3		4	4	196	211
**NISP totals**		**1057**	**1294**	**17,286**	**28,153**	**7976**	**8247**	**10,027**	**74,040**

**Fig 4:**
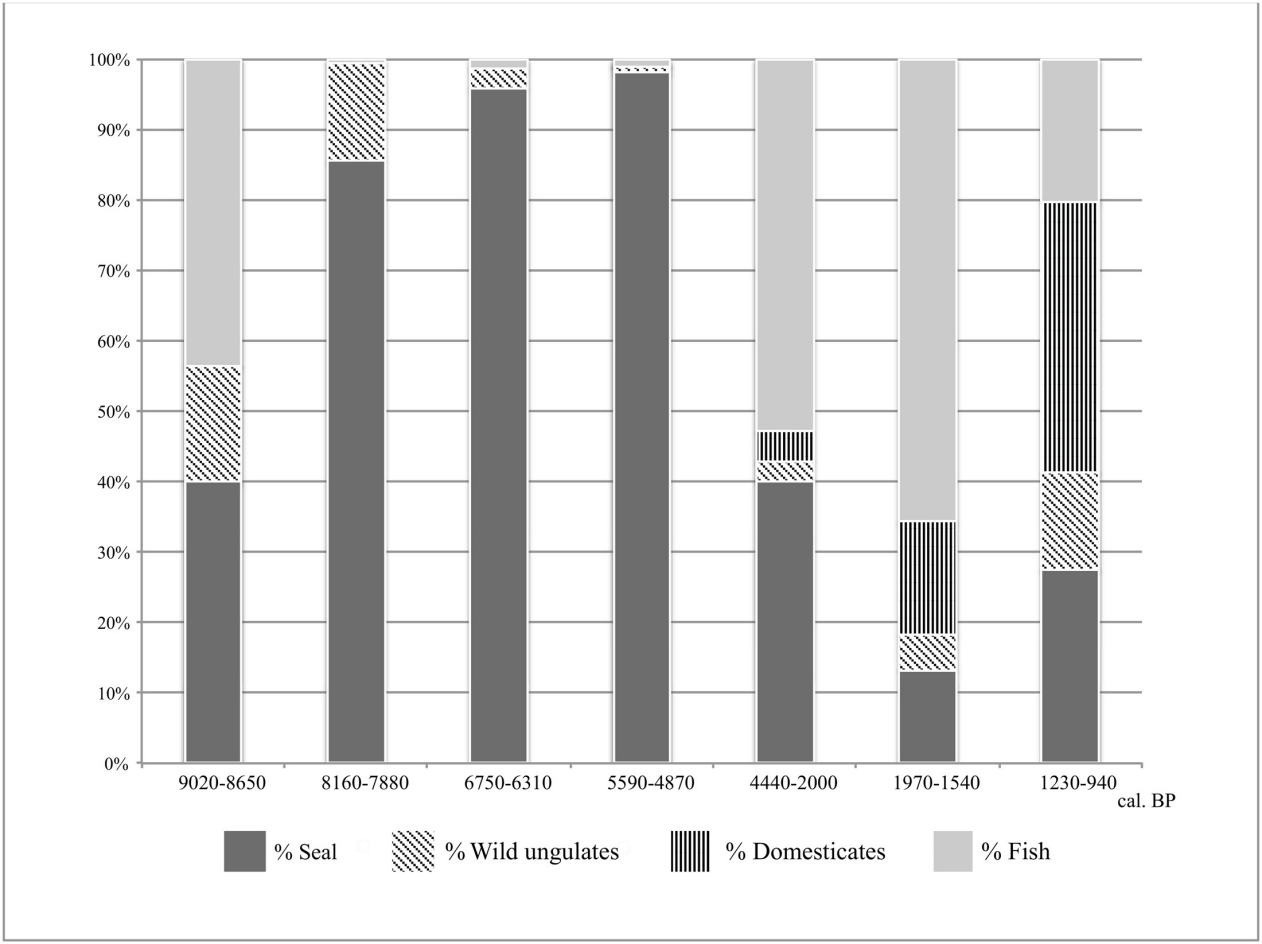
Plot of the relative abundances of seals, wild ungulates, domesticated ungulates, and fish at Sagan-Zaba II by analytical unit. NISP values for each group were used to calculate the percentages shown.

To explore how the abundance of seal remains changed over time and in relation to other categories of fauna, we use standard quantification measures such as NISP (Number of Identified Specimens), and MNI (Minimum Number of Individuals; following [66]). In addition, we present deposition rate estimates (Table 3; [40]), which are the number of faunal specimens recovered per estimated year of occupation for a given analytical unit. These are calculated by dividing NISP values for a layer by the maximum value of the ETI for that layer as presented in Table 1. These rates are used to assess the intensity of seal bone deposition at the site. To demonstrate the relative importance of seal remains at the site through time compared to those of other fauna, Fig 4 compares the relative abundances (by % NISP) of the four main taxonomic groups at the site, namely fish, wild ungulates (primarily red deer and roe deer), domesticated ungulates (sheep, goat, Caprinae family, and genera *Bos* and *Equus*), and seals, by analytical unit. Only specimens identified to at least the family level are included. Table 3 summarizes some of the key data from Fig 4.

**Table 3:** Summary of faunal data for Sagan-Zaba II including deposition rates of seal remains.

Seal Bone Estimates	Layers						
	VII	VI	VB-A	IVB-A	IIIB	IIIA	II-I
Seal NISP	101	296	6644	8540	416	112	174
Seal MNI	5	9	59	79	10	5	8
%Seal of total NISP to Order level^1^	59.4	82.9	94.3	98.2	42.8	12.8	24.5
Seal deposition rates (ETI seal dates)	0.4	1.3	21.5	8.3	0.1	n/a	n/a
Seal deposition rates (ETI ungulate dates)	0.4	3.7	18.1	9.2	0.2	0.1	1.2
All faunal deposition rates (ETI ungulate dates)	1.5	4.5	19.1	9.4	0.4	0.1	5

^1^Ground squirrel and families of mice and sparrows were excluded from calculations, as they are possibly intrusive taxa.

To explore the potential differential effects of taphonomic factors on the preservation of faunal remains at the site, we first visually evaluated the collection. The overall state of preservation was assessed as relatively good in all layers, with the exception of stratum IVA, where some the skeletal elements displayed grainy and irregular surfaces, likely indicating post-depositional erosion [40]. This may be attributable to the partial swamping of the deposits that occurred during formation of the layer [48]. Second, we tested for correlations between bone density and seal skeletal element survival from layer to layer. This was done by assessing element part abundances (using minimal number of elements (MNE) and minimal animal unit values (MAU)) with bone density values calculated for the Phocidae family [67]. The Phocidae values were used because specific density values for Baikal seals do not exist (detailed calculations are provided in [40]). These analyses showed that bone density measures and seal element part representation values were positively correlated in all layers (Spearman’s rho values ranged from 0.545 to 0.864). It has been shown, however, that variability in bone density alone cannot fully account for differences in skeletal element part survival in all depositional settings [68], and such correlations should be evaluated cautiously. Further caution is warranted because bone density values utilized here were not calculated on Baikal seal skeletal elements, which might differ from those of other members of the Phocidae family. Nonetheless, we believe the positive correlations between density values and element part survival observed at the site do indicate significant post-depositional attrition at Sagan-Zaba II. However, these data provide no indication that preservation conditions are radically different from layer to layer, particularly in terms of differential preservation of select taxonomic groups or age classes, which are our primary topics of interest.

The earliest evidence for seal use at Sagan-Zaba II is found in layer VII, which dates to ~9000 cal. BP (Tables 1 and 2). Seal remains here comprise almost 60% of the faunal remains identified to at least the order level, but the deposition rates for all fauna, including seal, are relatively low (Table 3). In layer VI, which was deposited ~8000 cal BP, seals account for 83% of the identified specimens, and the deposition rate also increased during formation of this layer. During the Middle Holocene, NISP and MNI values, as well deposition rates at Sagan-Zaba II all increase (Table 3). Seal remains constitute over 94–98% of the identified faunal remains in layers VB-A and IVB-A, and the seal deposition rate in layer V is many times higher than in the earlier dating layers, and far higher during these periods than in any other portion of Holocene represented in our sample. This increase could be due to either a higher frequency of visits to the cove or the taking of more seals per visit in comparison to earlier periods. The deposition rate decreases in layer IV, but is still far higher than in the younger layers. In layer IIIB, which spans from ~4440 to 2000 cal. BP, the actual and relative quantities of seal remains continue to decrease significantly, as do the deposition rates for remains of seals and other fauna (Table 3). Seal deposition rates rebound slightly in layers II-I, but still remain well below that seen in the Middle Holocene layers, while the overall rate of fauna deposition also increases compared to the preceding two layers. In layers II-I, seals remains constitute around 25% of the assemblage.

The dominant taxonomic groups at the site, namely seals, wild ungulates, domesticated ungulates, and fish show several shifts in relative abundances during the course of site occupation (Fig 4). The small assemblage from layer VII shows relatively high percentages of wild ungulates and fish, but these two groups of fauna decline in abundance relative to remains of seals into the Middle Holocene (layers VI, V, and IV), when the site was almost exclusively used for sealing. Layer IIIB, representing site use from 4440 to 2000 cal. BP shows a clear decline in seal remains (including a greatly reduced deposition rate), and exhibits a clear increase in the relative number of fish remains present compared to earlier levels, while wild ungulates remain a very minor component of the assemblage. Domesticated ungulates appear in this layer in trace quantities but then increase in relative abundance through the later two Late Holocene layers (after ~2000 cal. BP). Seals are at the lowest absolute and relative numbers in layer IIIA, which is instead dominated by remains of fish, followed by domesticates and wild ungulates, respectively. The younger strata (layers II-I, dating from 1230–940 cal. BP) show an increase in seal numbers compared to IIIA, while numbers of domesticates and wild ungulates also increase; fish remains are at their lowest relative abundance in the Late Holocene in these two layers.

## Seal Demographic Profiles

**Fig 5:**
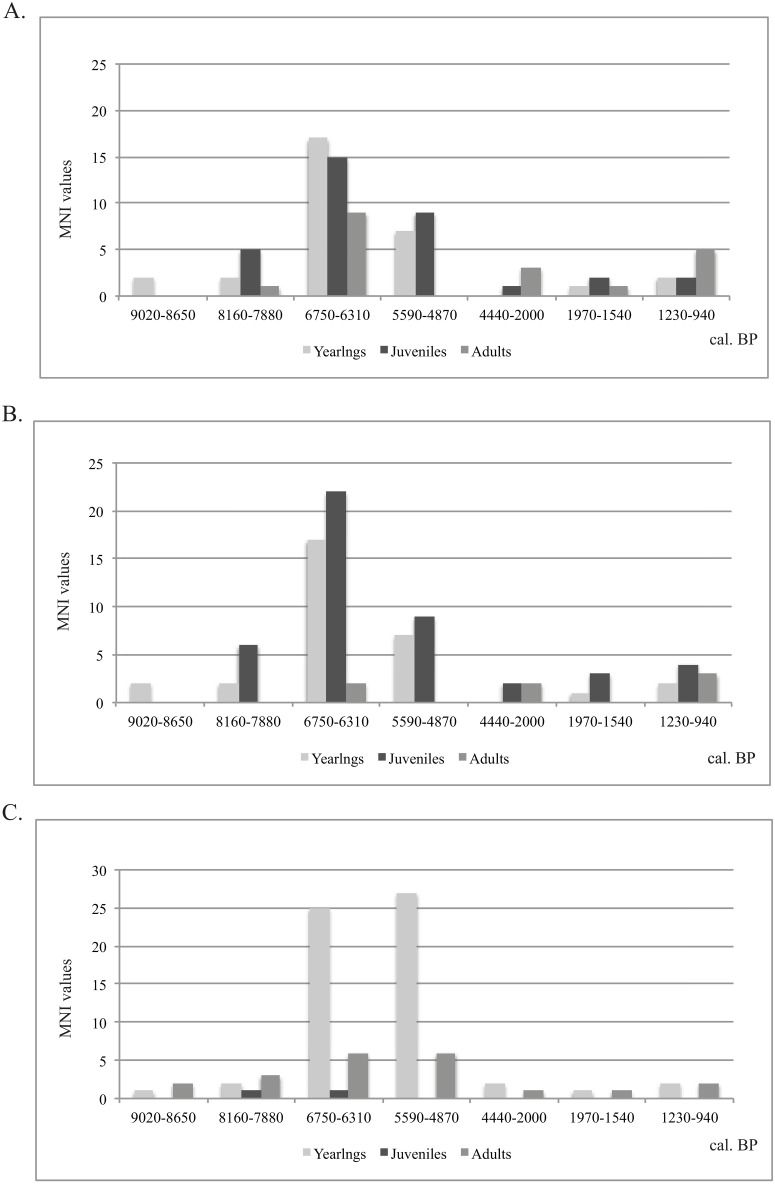
Seal age groups at Sagan-Zaba II. A: age groups based on canine incremental structures using female age of maturity; B: age groups based on canine incremental structures using male age of maturity; C: age groups based on epiphyseal fusion of long bones.

Two methods were used to reconstruct age at death for the seals represented at Sagan-Zaba. These are analyses of the incremental dentine bands in thin-sectioned canines and epiphyseal fusion of long bones. Dentine incremental structures of pinnipeds are widely accepted as a reliable technique for age determinations with an average error of +/- 1 year [69, 70]. Weber et al. [22, 45] developed this technique specifically for archaeological Baikal seal remains, and we apply his methodology here. Specifically identified canines (upper right canines, for example) from each analytical unit were selected for sectioning, with the total sample from Sagan-Zaba being 82 specimens. This was done to ensure that individuals were not sampled more than once. Where possible, dentine band age values were compared to those obtained from counting cementum bands, which allows for a check on the original age estimation. Female Baikal seals mature on average at four years of age, the males at six years, but their canines and mandibles cannot be identified to sex. Fig 5 provides the age assessments grouped into the age categories yearling (<1 year), juvenile, and adult. Note that Fig 5A shows the age groups assessments using the female maturity age, while Fig 5B uses the male maturity age.

Assessment of epiphyseal fusion is a relative ageing technique that involves assigning long bone elements to general age categories. This method has been developed for members of the *Phoca* genus by Storå [71] and previously applied to archaeological seal remains [14, 72]. These studies show that seals in *Phoca/Pusa* genera share similar sequences of epiphyseal fusion [71]. We assume these patterns are applicable also to *Phoca sibirica*, which are, as mentioned, genetically and morphologically similar to the ringed seals analyzed by Storå. In. Fig 5C, MNI values for each age category based on epiphysis fusion patterns are presented.

Seal age groups at the site reconstructed using both methods are compared in Fig 5. Several trends in the data are apparent. First, during the earliest and latest periods of occupation, when relatively few seals are represented at the site (from 9020 to 7880 cal. BP and after the 4440 cal. BP), there is no single age category that dominates—seals from all age groups were taken in similar quantities. The thin section data suggest a slight preference for juveniles in the earlier layers, while the late layers have relatively more adults, but the MNI values are small; the epiphysis fusion data also are probably too small to provide meaningful insights for these layers. The Middle Holocene (6750–4870 cal. BP) data, which in all cases include far larger numbers of individuals, show a more selective approach to seal hunting, with the focus clearly on yearlings and juveniles, with only a few adults represented. The thin section data point to a somewhat even focus on yearlings and juveniles, while the epiphysis fusion data indicate yearlings as the dominant category. The epiphysis ageing method, however, is better at identifying specimens as yearlings or as full adults, so these differences are in part an artifact of the method used. Regardless, both methods indicate an overwhelming focus on young seals during the Middle Holocene.

Sex assessments are difficult, as Baikal seals exhibit only minor sexual dimorphism in their crania [73], and no complete crania were recovered at the site. No information is available regarding sexually dimorphic characteristics in other skeletal elements. The only remaining skeletal evidence for seal sex assessment, thus is the presence or absence of bacula (i.e., *os penis*). Remarkably, only two small bacula fragments identified among the 16,000 seal remains at the site, and both were from juveniles in the site’s Middle Holocene layers. This could indicate the nearly complete absence of male seal remains at the site, but we believe that this conclusion is likely incorrect. The overall seal assemblage is dominated by yearlings and juveniles, and seals of this age are not visually sexually dimorphic and thus would be extremely difficult for even experienced hunters to differentiate [33]. The very low number of bacula from young seals is more likely related to preservation—these elements are partially ossified at this life stage and thus are unlikely to survive. However, we do believe the near-total absence of bacula at the site indicates that the majority of the adults are females rather than males, as the bacula of adult male seals are very distinct and quite robust. Furthermore, adult male seals are not highly prized by contemporary hunters on the lake due to their pungent smell and taste (and are readily differentiated from adult females; [33]), and modern seal distribution patterns [30] indicate that adult males are not likely to have been the predominant age group near Sagan-Zaba during any season of the year.

## Seal Hunting Seasonality

**Fig 6:**
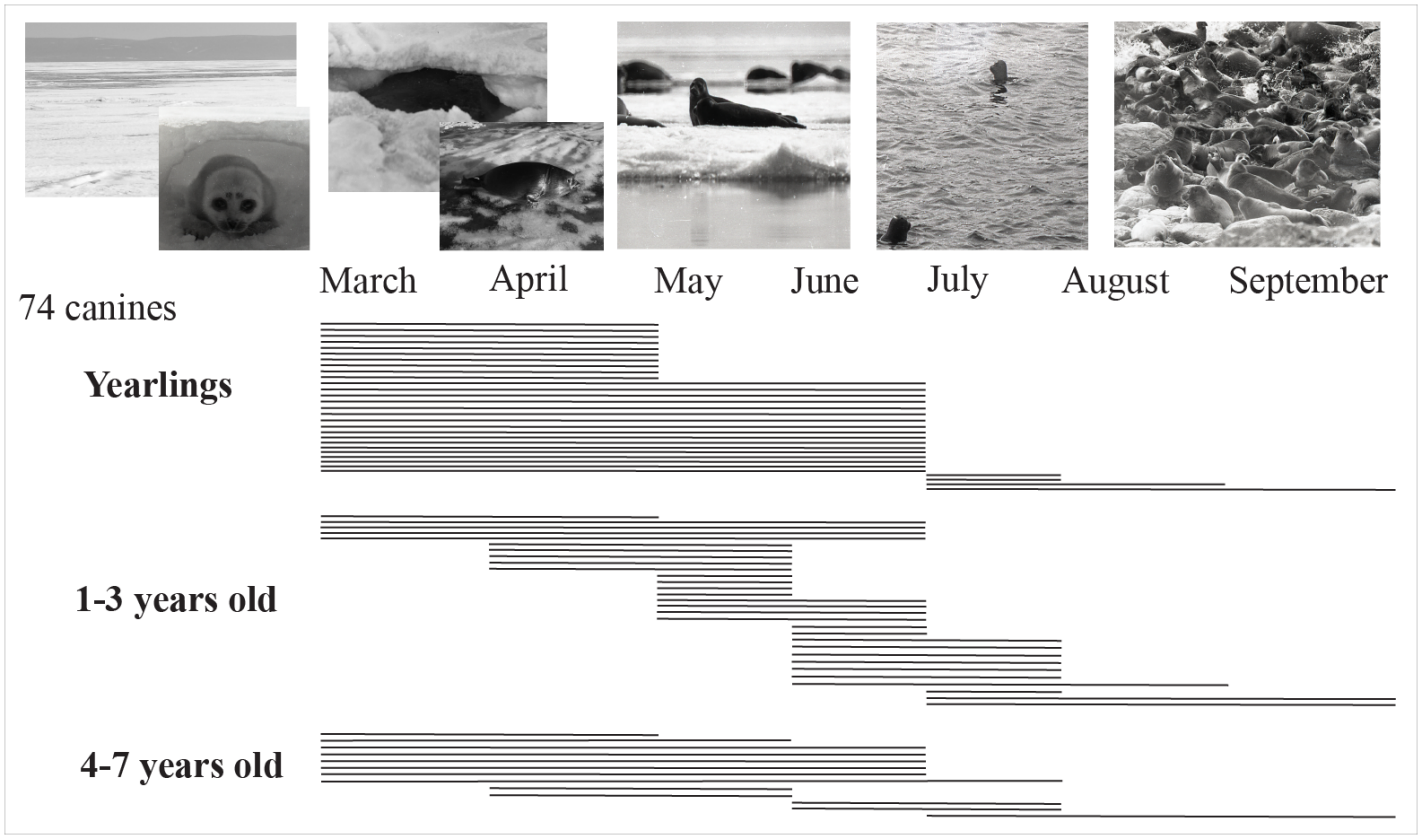
Seal seasonality data at Sagan-Zaba II by age groups (photographs by V.D. Pastukhov). Each bar represents a season of death estimation for a single individual.

**Fig 7:**
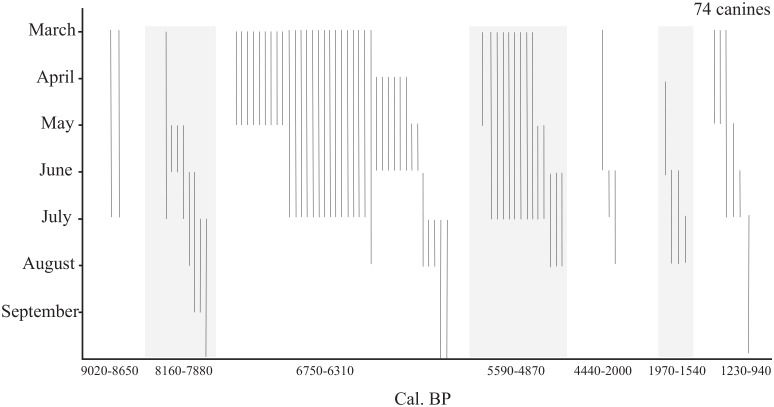
Seal seasonality data at Sagan-Zaba II by time period. Each bar represents a season of death estimate for a single individual.

Assessment of the seasonality of seal use is based on season of death estimations made on incremental dentine bands in 74 canines, following the method established by Weber et al. [45] for Baikal seals. The estimations were made on the same canine thin sections as described above, with eight specimens being too poorly preserved to be reliably read. Overall, these analyses suggest that seals found at Sagan-Zaba II were taken from March to September, with most killed in the spring, and only a few in the summer months (Fig 6). Layer VII, the earliest at the site, dating to 9020 to 8650 cal. BP, contained only two specimens that could be assessed, and these were assigned to the spring and early summer period (Fig 7). Layer VI, dated to the very earliest part of the Middle Holocene, had specimens from seals that potentially died throughout the seven month period, but most appear to have been taken from late spring through summer. Layers V and IV represent the periods of most intensive seal use at the site, and in the earlier of the two (layer V, from 6750 and 6310 cal. BP) sealing appears to have peaked around April, while in the following period (IV, from 5590 to 4870 cal. BP), no such clear peak is in evidence, but the season of death estimates generally spans spring and very early summer. The three Late Holocene layers also generally fall within the spring, but a few individuals may be from the summer months.

When the seasonality data are viewed in conjunction with the age assessments and observations of modern seal behaviour (Fig 7), it appears that most of the yearlings were taken from March to April, with only a few such individuals taken in the following months of the year. From March through April the lake is typically frozen over and the yearlings inhabit their dens. They are particularly vulnerable towards the end of this period in April, when the dens begin to melt open with the season’s rising temperatures, and which is also marked by these animals regularly sunbasking along the margins of developing ice-free areas. This period also is the main sealing season on Lake Baikal in the historic and modern periods, when hunters too focused largely on procuring yearlings [33]. Further, a spring focus on yearlings also was suggested in previous investigations of seal remains from several Baikal archaeological sites [22, 45].

Juveniles (1–3 year olds) were mainly hunted from May to June, with a few taken in summer. Seasonality of death for the young adults (4–7 year olds) falls mainly in the spring, just like that seen with the majority of the yearlings, and it is possible that these individuals were nursing females, which would accompany the yearlings during this period of their development. A few younger adults also were taken at the beginning of summer, and these individuals may have been taken on the ice following the period of moulting, which is generally completed by seals of all age categories by the beginning of June. Some seals, particularly juveniles, likely were taken during the open-water season (June to September), when the seals intensively feed. It is also the only time of the year in which the seals regularly bask along the lake’s rocky shorelines [30], and perhaps the shore of Sagan-Zaba cove and that of the adjoining cliffs were summer haulouts for seals in the past.

Overall, the seal season of death data fairly clearly suggests that human occupation of Sagan-Zaba cove occurred mostly in spring with some use also occurring in summer and early fall. This data of course cannot exclude human use of the cove during other seasons. Notably, other seasonal indicators, such as the trace quantities of migratory bird remains (e.g., cormorants, ducks) at the site, also support the most use of this location during the warmer periods of the year.

## Seal Bone Modification and Associated Materials

Seal remains at Sagan-Zaba II were found in direct association with other archaeological materials such as portable artifacts and features, and some skeletal elements from all layers were modified, all providing clear indication that humans were responsible for their accumulation at the site. No evidence of living structures was found at the site, but many features were present in all site layers, each being either simple hearths, or concentrations of faunal remains, other artifacts such as ceramics, or unmodified stones. Some of the more significant features are described below.

Seal skeletal elements from all layers displayed butchery marks and traces of burning. While in layers VII through IIIB the percentages of seal specimens with cut marks is relatively low, ranging from 0.2–0.8%, the Late Holocene layers show far higher relative numbers of specimens with cut marks: 2.7% and 5.2% in layers IIIA and II-I, respectively. The relatively low number of seal remains with cut marks is perhaps not surprising given the fact that yearlings and juveniles dominate the seal assemblage at the site. Historically, yearlings weigh no more than 31 kg by the fall, and juveniles anywhere from 34–58 kg [30]. In other words, their bodies perhaps were not large enough to require extensive field processing to facilitate transport, nor would they have to be extensively disarticulated prior to cooking. Notably, in all site layers, other identified mammal remains showed higher percentages of cut-marked specimens than seen among their respective seal remains; the majority of these taxa are far larger bodied than Baikal seals [40]. Burning traces also were observed in all layers at the site, ranging from a high of 14.9% of specimens in layer VII to as low as 1.2% in layer VA-B. It is unknown to what extent these traces are indicative of cooking activities at the site, use of seal body parts as fuel, discard of unused portions into hearths, or incidental burning due to hearths being placed near previously discarded bones.

**Fig 8:**
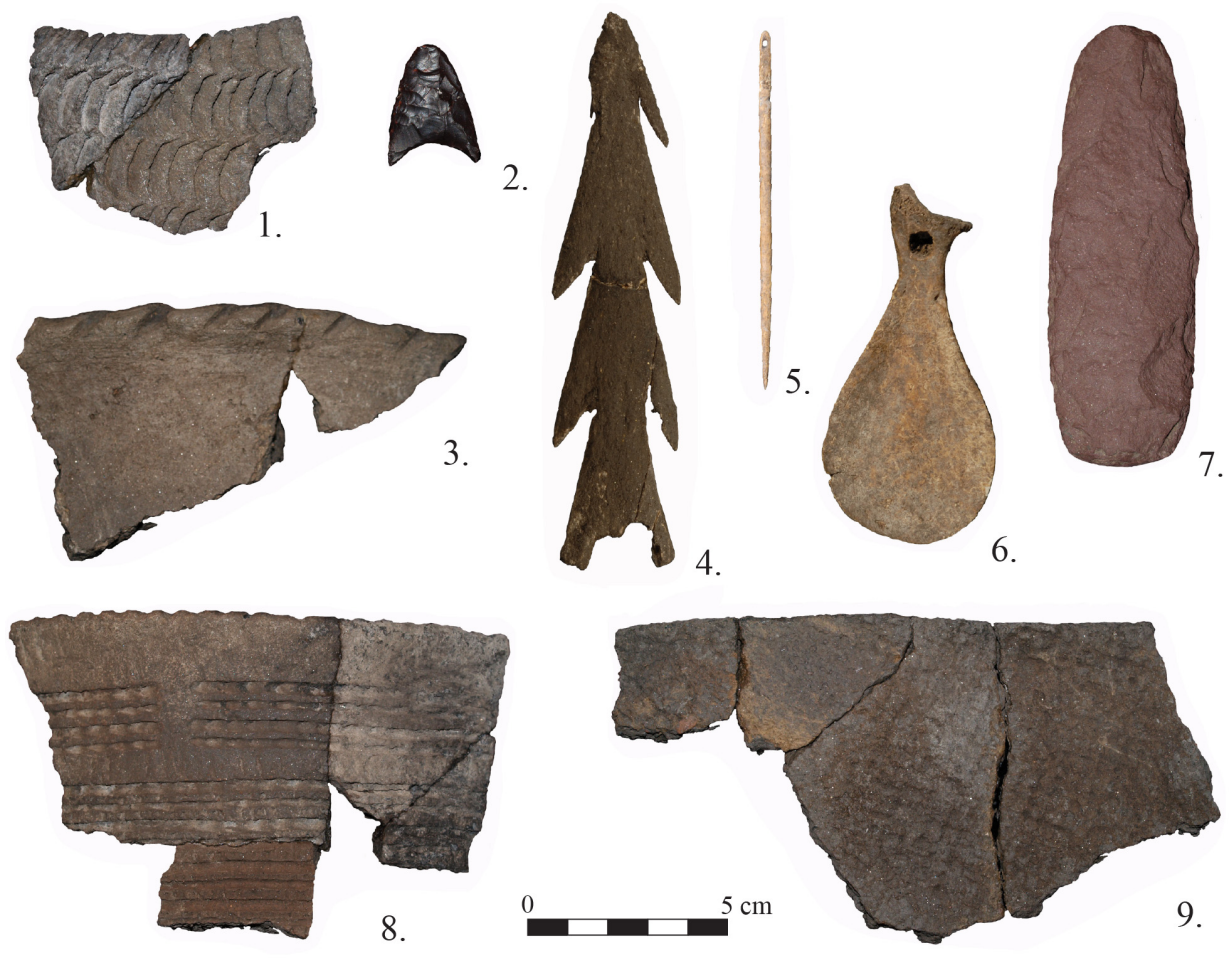
Examples of artifacts found at Sagan-Zaba II. Numbers 1 and 3 are from Late Holocene layers IIIB-I; 2, 5, and 7 are from Middle Holocene layers V-IV; 4 and 9 are from Early to Middle Holocene layers VII-VI (photographs by T. Nomokonova).

In the earliest site layers (VII and VIB-A), seal remains were mostly found near hearths and simple stone features consisting of small piles of colluvium. Other artifacts from layer VII consist of 86 stone and bone items including microblades, prismatic stone inserts for bone tools, burins, a scraper, an abrader, and a quartzite sinker. Among the few bone implements from the layer are fragments of a harpoon head (Fig 8: 4), a point, and fragments of insert tools. Layers VIB-A contained 769 portable artifacts including 602 ceramic fragments, 151 stone items, a few bone tools and small shell beads. Stone tools and debitage were present, the former primarily consisting of microblades but scrapers, inserts, adze-like tools, burins, reamers, and a shank of a composite fishhook also were present. Bone tools include a needle, harpoon head, spoon, and insert tools [64, 74].

**Fig 9:**
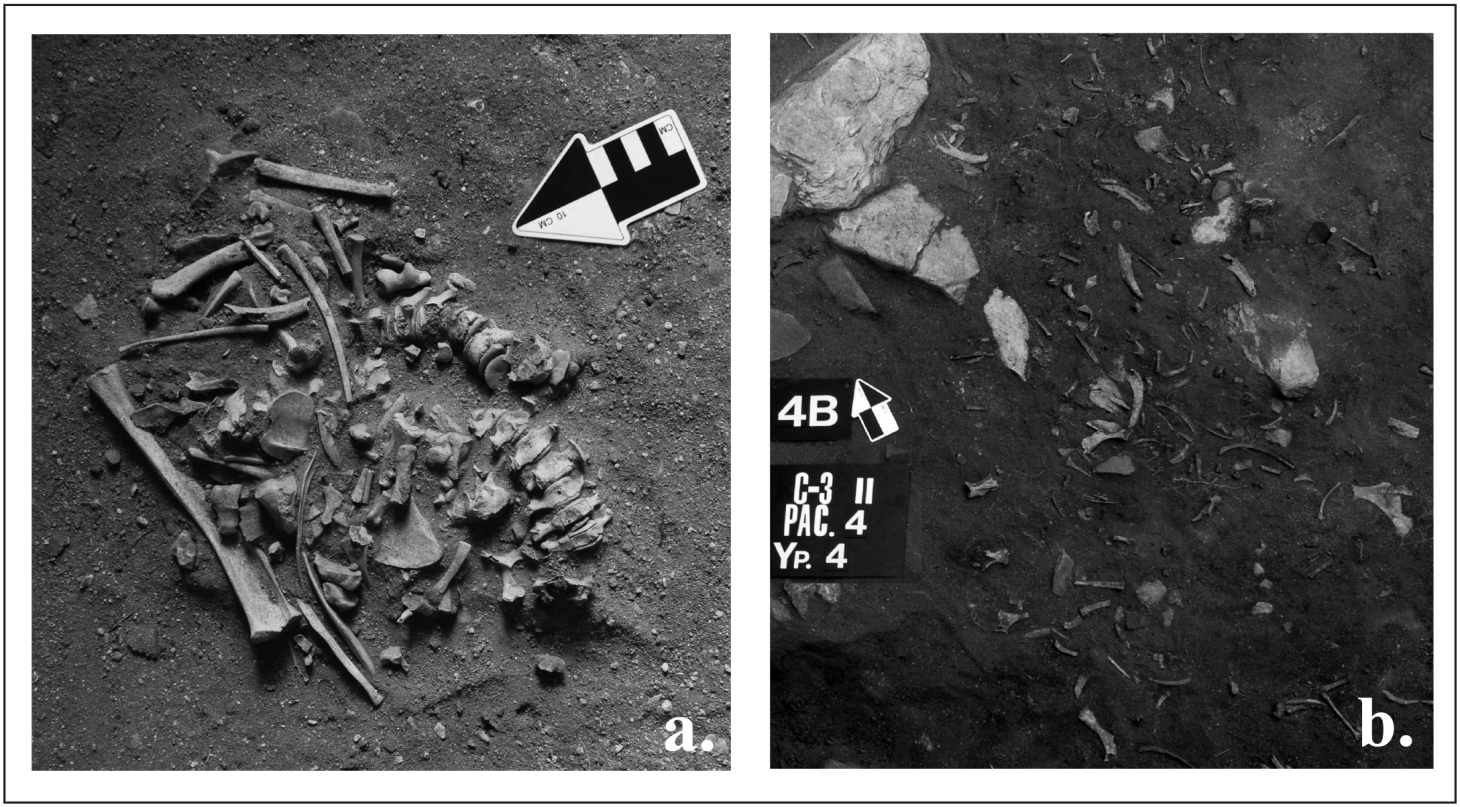
Example of seal bone clusters found at Sagan-Zaba II (a—layer VB, b—layer IVA). Photographs by A. Weber.

Middle Holocene layers VB-A and IVB-A are very distinct in terms of spatial patterning of seal remains when compared to other site layers. These layers contained multiple features consisting largely of dense clusters of seal bone from multiple individuals, with the elements being disarticulated, the exception being a few cases of articulated vertebrae (Fig 9). For example, layer VB had four clusters, some in close association with stone features and hearths (Fig 9a). These clusters contained two to 16 individuals each. Layer IVA had only a single large (2.8 x 1.4 m) cluster that produced remains of at least 48 seals (Fig 9b). Typologically similar artifacts were found in layers V and IV. The bone clusters and the surrounding sediments in both layers contained pottery fragments, lithic and bone tools, and other faunal remains. These include 1230 stone items, 1794 pottery fragments, and 35 bone implements. Stone items consisted of scrapers, arrowheads (Fig 8: 2), knifes, adze-like items (Fig 8: 7), burins, borers, prismatic blade inserts, shanks of a composite fishhooks, and a fragment of stone fish lure. Bone items include harpoon heads, arrowhead, tools for lithic inserts, needls (Fig 8: 5), points, a spoon, beads, and pendants [64, 75–76].

No bone cluster features were present in the site’s late Holocene layers. Layer IIIB contained 796 stone artifacts, including scrapers, arrowheads, borers, drills, a knife, and a prismatic blade insert, and 514 pottery fragments also were present. Osseous tools present were a bone needle case, antler point, harpoon fragment, and an arrowhead; a single bronze awl also was present in the layer. The Late Holocene artifacts from layers IIIA and II-1 contained many metal items such as simple iron awls, and a Chinese bronze coin and a finger ring also were recovered. Bone items from the layers consist of a spoon made from a seal scapula (Fig 8: 6), three modified astragals, and a few stone flakes and scrapers (the latter present in layer IIIA only). These layers also contained 645 pottery fragments [27, 63].

## Discussion

Sagan-Zaba cove provides ample evidence of seal hunting throughout much of the Holocene, and within this time span several trends are apparent. First, sealing began here at least 9000 years ago, which to our knowledge is the earliest clear evidence for such practices in North Asia. This Early Holocene onset of sealing at Sagan-Zaba is consistent with the previously existing evidence for early seal hunting at Lake Baikal, which consisted of only a handful of remains, also apparently dating to the Early Holocene [39, 40, 44]. At Sagan-Zaba II, this hunting probably occurred in the spring on the lake ice, and at even this point in the history of the site, seal remains were relatively abundant, suggesting sealing was a primary subsistence activity here at this time. It remains unclear if the advent of sealing on Lake Baikal marks an expansion of dietary breadth or some other shift in adaptive strategies among local foragers here during the Early Holocene. As indicated above, there is no inherent reason why sealing could not have occurred at Lake Baikal in the Pleistocene, but the near-total absence of Pleistocene sites along the lake’s shores currently makes comparisons with earlier periods impossible. We speculate that changes in lake level during the Holocene may have eroded or stranded Pleistocene sites along the shore of Baikal, hiding or erasing evidence for the earliest use of seal at Lake Baikal.

Second, the peak of seal use at the site is the Middle Holocene (layers V and IV), when seals overwhelming dominate the faunal assemblages (> 90% of the NISP) and were deposited at far higher rates than observed during any other period of site use. In this period the foragers occupying Sagan-Zaba appear to have been seasonally specialized seal hunters. This sealing in many ways mirrors that seen in the region during the historic period [33]. Namely, hunters focused their efforts on yearlings and juveniles that were taken in the spring on the lake ice. Both historically and in our experience with seal hunters on the lake [33], seals of this age are and were chosen because they are relatively fat-rich, less wary than the adult females, and taste and smell better than adult males, which are more easily approached than adult females but are nonetheless not highly regarded. The warming temperatures of spring in particular provide the seals with the opportunity to sun bask on the melting lake ice, which renders them quite visible and more easily accessible than in other seasons, particularly compared to the open water period [33]. It seems likely that these same factors were influencing sealing patterns on Lake Baikal during the Middle Holocene. Stable carbon and nitrogen isotope analyses of human remains from the Little Sea region, only about 50 km to the north of Sagan-Zaba II, also suggest that many Middle Holocene people in this region consumed Baikal seal and its fish [19, 24]. However, the vast majority of these individuals date to the Early Bronze Age, with earlier portions of the Holocene being represented by a total of only 12 skeletons. The Sagan-Zaba data clearly show that seal use predates the bulk of this mortuary evidence, and helps to fill some gaps in it.

The peak in sealing at Sagan-Zaba appears to have begun at ~6750 cal. BP, almost precisely at the same time as the broader region experienced a discontinuity in the use of formal cemeteries during the Middle Neolithic (~6800 to 5700 cal. BP; [54]). This discontinuity followed a period of significant climate change in the region, which involved the onset of drier and colder conditions beginning around 7000 cal. BP [77–83]. The effects of this climate change on the lake’s seals are unknown, but the faunal data from Sagan-Zaba II provide no indication of a detrimental impact upon them. Further, the faunal data from Sagan-Zaba provide no support for argument that sealing was most intensive at Lake Baikal during the Bronze Age (~5000 to 3400 cal. BP; [22, 45]). Layer IIIB, which dates to 4440–2000 cal. BP, shows a marked decrease in the deposition rate of seal remains and a clear increase in the relative abundances of other taxa, particularly fish and ungulates. A similar pattern also is present at the Tyshkine II/III habitation sites (on Ol’khon Island) about 75 km northeast of Sagan-Zaba, where seal remains are more abundant in the layers predating the Bronze Age [60]; this site, however, is not well dated, and the collections are now lost. Notably, the Bronze Age may also witness the creation of the petroglyphs at Sagan-Zaba [62, 84–85], which could further indicate a change in the use and meaning of this place.

Third, the other major shift in subsistence practices at the site occurs in the Late Holocene with the introduction of domesticated herd animals, which first appear in layer IIIB, or some time between 4440 and 2000 cal. BP. Remains of fish and wild ungulate also are relatively more common at the site following the Middle Holocene. Remains of domesticates are initially a very small component of the faunal assemblage, and only become relatively numerous some time after ~2000 cal. BP. This pattern closely follows that seen at the Bugul’deika II habitation site, located about 40 km southwest of Sagan-Zaba along the open shoreline of the lake. At Bugul’deika, the earliest directly dated remains of domesticates (a horse), are ~2800 cal. BP old, but only after ~2000 cal. BP are such animals abundant at the site [38–39]. The 2800 cal. BP date predates by a few centuries the radiocarbon dates on the earliest pastoralist graves in this region of the western lake shore [26, 86]. Analysis of cores taken from Lake Khall, located 3 km inland from Sagan-Zaba, shows that the local region experienced rapid climate change from ~2750 to 2500 cal. BP involving a marked drop in precipitation, and a decline in the mean temperature in the coldest month and an increase in the mean temperature of the warmest month; these changes were associated with shifts in forest composition, including the increasing abundance of drought-resistant Scots pine (*Pinus sylvestris*; [87]). A second period of severe droughts affected the area between ~2100 and 1900 cal. BP, which was followed by a period of relative stability characterized by warmer summer temperatures and increased forest cover, resulting in less steppe vegetation [87]. How these climate and environmental transitions relate to the changes in subsistence practices observed at Sagan-Zaba II and Bugul’deika II, and the local area’s Late Holocene culture history more generally, is presently unclear and needs further exploration. Broad socio-political changes and migrations in adjacent Central Asia also may have played a role in the region’s pastoral histories [27].

Importantly, the Late Holocene deposits at both Sagan-Zaba II and Bugul’deika II contain some remains of seals and fish, indicating that these pastoralist groups also procured and consumed these animals, which is entirely consistent with historical practices of herding groups from this region [33]. Subsistence economies in the region near these sites during the Late Holocene clearly were a mix of herding, hunting of seals and terrestrial mammals, and also fishing. Documenting the past use of the lake’s aquatic fauna by local herding groups is important, as these animals carry a significant old carbon offset making resulting ^14^C dates substantially older than their real age [47]. The culture history of the region’s pastoralist cultures, which is built in part upon radiocarbon dates on human skeletal remains, likely will need correction for this old carbon offset, as has recently been done for the region’s Middle Holocene forager mortuary sites [54].

## Conclusions

Examination of Sagan-Zaba’s faunal assemblage, including its chronology, association with other artifacts, and relations to climate and environmental changes, have provided a series of insights on sealing practices on Siberia’s largest lake. Some seal hunting first occurred at this site at least 9000 years ago, and by the Middle Holocene was regularly practiced from Sagan-Zaba cove, when foragers focused their efforts on procuring young seals basking on the spring ice. Seals were butchered and consumed at the site during all periods of occupation, including by pastoralists groups, who first utilized the cove some time just prior to 2000 cal. BP. Fishing and hunting of terrestrial fauna, mostly ungulates, were relatively minor activities at Sagan-Zaba II during the Middle Holocene period, when the site was most frequently utilized—sealing clearly was the predominant subsistence activity during this period. Fishing was relatively more important during the earliest periods of site use, and again in the Late Holocene, the latter period also witnessing the increasing importance of ungulate game hunting when compared to the Middle Holocene.

Future research on sealing at Lake Baikal will undoubtedly have to come to terms with how this record was affected by a series of increasingly well-documented climate shifts, episodes of human migration and demographic change, and fluctuations in sociopolitical and ideological systems. This paper is one important step in this direction. Finally, it is clear that humans’ adaptations to Lake Baikal aquatic resources have a temporal depth that rivals that of some of the world’s marine coastal regions, particularly in terms of the antiquity of intensive pinniped use [88]. Lake Baikal provides a truly unmatched environment in which to study the long-term history human aquatic adaptations due to its extreme age and unique fauna. We hope our research on seal hunting at Sagan-Zaba provides additional motivation for such explorations.

## References

[1] Balikci A. The Netsilik Eskimo. Garden City, New York: The Natural History Press; 1970.

[2] Boas F. The Central Eskimo Sixth Annual Report of the Bureau of Ethnology. Washington, D.C.: Smithsonian Institution; 1888.

[3] Bogoras W. The Chukchee Memoirs of the American Museum of Natural History, Vol. 11, Parts 1–3 Leiden, New York: E.J. Brill ltd and G.E. Stechert & Co, Leiden; 1904–1909.

[4] Braje TJ, Rick TC, editors. Human Impacts on Seals, Sea Lions, and Sea Otters: Integrating Archaeology and Ecology in the Northeast Pacific. Berkeley: University of California Press; 2011.

[5] Diab MC. Economic utility of the ringed seal (*Phoca hispida*): implications for Arctic archaeology. J Archaeol Sci 1998; 25: 1–26.

[6] Edlund AC. The Swedish seal-hunters’ conceptual system for seal: a cognitive, cultural and ecological approach In Soares da Silva A, Torres A, Gonc¸alves M, editors. Linguagem, Cultura e Cognic¸ao: Estudos de Linguistı´ca Cognitive. Coimbra: Almedina, Coimbra; 2004 p. 215–229.

[7] Juel E. Notes on seal-hunting ceremonialism in the Arctic. Ethnos 1945; 10: 143–164.

[8] Kreinovich EA. Morskoi promysel Giliakov d. Kul’. Sovetskaia Etnografiia 1934; 5: 78–96. Russian.

[9] Monks G, editor. The exploitation and cultural importance of sea mammals Proceedings of the 9th ICAZ Conference, Durham 2002. Oxford: Oxbow Books; 2005.

[10] Nelson R. Hunters of the Northern Ice. Chicago: University of Chicago Press; 1969.

[11] Nuttall M. Arctic Homeland Kinship, Community and Development in Northwest Greenland. Toronto: University of Toronto Press; 1992.

[12] Ohtsuka K. Nivkh seal hunting and ritual. B Natl Mus Ethnol 1994; 19: 543–585.

[13] Pelly DF. Sacred Hunt A Portrait of the Relationship Between Seals and Inuit. Seattle: University of Washington Press; 2001.

[14] Storå J. Neolithic seal exploitation on the Aland Islands in the Baltic Sea on the basis of epiphyseal fusion data and metric studies. Int J Osteoarchaeol 2002; 12: 49–64. 11913358

[15] Dikov NN. Arkheologicheskie Pamiatniki Kamchatki, Chukotki i Verkhnei Kolymy: Aziia na Styke s Amerikoi v Drevnosti. Moscow: Nauka; 1977. Russian.

[16] Kiryak (Dikova) MA. The Stone Age of Chukotka, Northeastern Siberia (New Materials) BAR International Series 2099. Oxford: British Archaeological Reports; 2010.

[17] Kuzmin YV. Paleoeconomy of the Russian Far East (Stone Age complexes) In: Nelson SM, Derevianko AP, Kuzmin YV, Bland RL, editors. Archaeology of the Russian Far East: Essays in Stone Age Prehistory. BAR International Series 1540. Oxford: British Archaeological Reports; 2006 p. 167–173.

[18] Petrov EA. Baikal’skaia Nerpa. Ulan-Ude: Belig; 2009. Russian.

[19] Katzenberg A, McKenzie HG, Losey RJ, Goriunova OI, Weber AW. Prehistoric dietary adaptations among hunter-fisher-gatherers from the Little Sea of Lake Baikal, Siberia, Russian Federation. J Archaeol Sci 2011; 39: 2612–2626.

[20] Nomokonova T, Losey RJ, Goriunova OI. Prehistoric Fishing on Lake Baikal, Siberia: Analyses of Faunal Remains from Ityrkhei Cove. Saarbrücken: VDM Verlag Dr. Mueller; 2009.

[21] Weber A. The Neolithic and Early Bronze Age of the Lake Baikal Region: a review of recent research. J Word Prehist 1995; 9(1): 99–159.

[22] Weber A, Link DW, Goriunova OI, Konopatskii AK. Patterns of prehistoric procurement of seal at Lake Baikal: a zooarchaeological contribution to the study of past foraging economies in Siberia. J Archaeol Sci 1998; 25: 215–227.

[23] Weber AW, Link DW, Katzenberg MA. Hunter-gatherer culture change and continuity in the Middle Holocene of the Cis-Baikal, Siberia. J Anthropol Archaeol 2002; 21: 230–299.

[24] Weber AW, White D, Bazaliiskii VI, Goriunova OI, Savel’ev NA, Katzenberg MA. Hunter-gatherer foraging ranges, migrations, and travel in the middle Holocene Baikal region of Siberia: Insights from carbon and nitrogen stable isotope signatures. J Anthropol Archaeol 2011; 30(4): 523–548.

[25] Kharinskii AV. Predbaikal’e v kon I tys. do n.e.—ser. II tys n.e.: Genesis Kul’tur i ikh Periodizatsiia. Irkutsk: IrGTU; 2001. Russian.

[26] Kharinskii AV. Zapadnoe poberezh’e ozera Baikal v I tys. do n.e.- I tys. n.e In: Kharinskii AV, editor. Izvestiia Laboratorii Drevnikh Tekhnologii. Volume 3 Irkutsk: IrGTU; 2005 p. 198–215. Russian.

[27] Nomokonova TIU, Losey RJ, Weber A, Goriunova OI, Novikov AG. Late Holocene subsistence practices among Cis-Baikal pastoralists, Siberia: zooarchaeological insights from Sagan-Zaba II. Asian Perspec 2010; 49(1): 157–179.

[28] Georgi IG. Opisanie vsekh v Rossiiskom Gosudarstve Obitaiushchikh Narodov, takzhe ikh Zhiteiskikh Obriadov, Very, Obyknovenii, Zhilishch, Odezhd i Prochikh Dostopriamichatel’nostei. Part 3 St. Petersburg: Tipografiia K.V. Miullera; 1777. Russian.

[29] Levin NP. Rybolovstvo i rybopromyshlennost’ na Ol’khone. Izvestiia VSORGO 1897; 28: 44–81. Russian.

[30] Pastukhov VD. Nerpa Baikala: Biologicheskie Osnovy Ratsional’nogo Ispol’zovaniia i Okhrana Resursov. Novosibirsk: Nauka; 1993. Russian.

[31] Toporkov NN. Ekonomicheskoe znachenie nerpich’ego promysla. Severnaia Aziia 1926; 5–6: 63–75. Russian.

[32] Zhambalova SG. Okhota na nerpu u Ol’khonskikh buriat In: Mikhailov TM, Nimaev DD, Rassadin VI, editors. Etnicheskaia Istoriia i Kul’turno-bytovye Traditsiii v Buriatii. Ulan-Ude: Akademiia Nauk SSSR p. 97–107; 1984. Russian.

[33] Nomokonova T, Losey RJ, Iakunaeva VN, Emel’ianova IA, Baginova EA, Pastukhov MV. People and Seals at Siberia’s Lake Baikal. Ethnobiol 2013; 33(2): 259–280.

[34] Weber AW, Bettinger R. Middle Holocene hunter-gatherers of Cis-Baikal, Siberia: an overview for the new century. J Anthropol Archaeol 2010; 29: 491–506.

[35] Weber AW, Goriunova OI, McKenzie HG, Lieverse AR, editors. Kurma XI, a Middle Holocene Hunter-Gatherer Cemetery on Lake Baikal, Siberia: Archaeological and Osteological Materials Northern Hunter-Gatherers Research Series 6. Edmonton: CCI Press; 2012.

[36] Weber AW, Katzenberg MA, Schurr TG, editors. Prehistoric Hunter Gatherers of the Baikal Region, Siberia: Bioarchaeological Studies of Past Life Ways Philadelphia: U Penn Museum Press; 2010.

[37] Losey RJ, Nomokonova T, Goriunova OI. Fishing Ancient Lake Baikal, Siberia: Inferences from the Reconstruction of Harvested Perch (Perca fluviatilis) Size. J Archaeol Sci 2008; 38: 577–590.

[38] Losey RJ, Nomokonova TIU, Savel’ev NA. Radiouglerodnoe datirovanie i fauna mnogosloinoi stoianki Bugul’deika II na Baikale (po materialam raskopok 2006–2008 gg.) Izvestiia IGU. Seriia Geoarkheologiia, Etnologiia, Antropologiia 2014; 7: 3–17. Russian.

[39] Losey RJ, Nomokonova T, Savel'ev NA. Humans and animals at Bugul'deika II, a Trans-Holocene Habitation site on the shore of Lake Baikal, Russia. Quatern Int 2014 doi: 10.1016/j.quaint.2014.08.021

[40] Nomokonova T. Holocene Sealing and Pastoralism at Sagan-Zaba Cove, Siberia [dissertation]. Edmonton (AB): University of Alberta; 2011.

[41] Katzenberg MA, Weber AW. Stable isotope ecology and palaeodiet in the Lake Baikal region of Siberia, J Archaeol Sci 1999; 26: 651–659.

[42] Katzenberg MA, Goriunova OI, Weber A. Paleodiet reconstruction on Bronze Age Siberians from the mortuary site of Khuzhir-Nuge XIV, Lake Baikal. J Archaeol Sci 2009; 36(3): 663–674.

[43] Katzenberg MA, Bazaliiskii VI, Goriunova OI, Savel'ev NA, Weber AW. Diet reconstruction of prehistoric hunter—gatherers in the Lake Baikal region In: Weber AW, Katzenberg MA, Schurr T, editors. Prehistoric Hunter—Gatherers of the Baikal Region, Siberia: Bioarchaeological Studies of Past Lifeways Philadelphia: University of Pennsylvania Press; 2010 p. 175–192.

[44] Nomokonova T, Losey RJ. Seal hunting in the Little Sea region of Lake Baikal, Siberia In: Hokkaido Museum of Northern People, Proceedings of the 27th annual international Abashiri symposium. Abashiri: The Association for the Promotion of Northern Cultures; 2013; p. 25–30.

[45] Weber A, Goriunova OI, Konopatskii AK. Prehistoric seal hunting on Lake Baikal: methodology and preliminary results of the analysis of canine sections. J Archaeol Sci 1993; 20: 629–644.

[46] Nomokonova T, Losey R, Goriunova OI, Weber A, Novikov AG, McKenzie H. Perspektivy zooarkheologicheskikh issledovanii v bukhte Sagan-Zaba na Baikale (po resul’tatam rabot 2006 g.) Vestnik NGU. Seriia: Istoriia, Filologiia 8 (5: Arkheologiia i Etnografiia); 2009 p. 116–122. Russian.

[47] Nomokonova T, Losey RJ, Goriunova OI, Weber AW. A freshwater old carbon offset in Lake Baikal, Siberia and problems with the radiocarbon dating of archaeological sediments: evidence from the Sagan-Zaba II site. Quatern Int 2013; 290–291: 110–125.

[48] Vorob’eva GA, Goriunova OI, Novikov AG, Weber AV. Arkheologicheskie i paleoekologicheskie aspekty obitaniia cheloveka na mnogosloinom geoarkheologicheskom ob”ekte Sagan-Zaba II (po materialam raskopok 2006 g.) In: Kharinskii AV, editor. Izvestiia Laboratorii Drevnikh Tekhnologii. Volume 7 Irkutsk: IrGTU; 2009 p. 73–85. Russian.

[49] Aksenov MP. K istorii issledovaniia dokeramicheskikh mestonakhozhdenii In Medvedev GI, Savel’ev NA, Svinin VV, editors. Stratigrafiia, Paleografiia i Arkheologiia Iuga Srednei Sibiri. Irkutsk: IGU; 1990 p. 88–100. Russian.

[50] Goebel T. Pleistocene human colonization of Siberia and peopling of Americas: an ecological approach. Evol Anthropol 1999; 8(6): 208–227.

[51] Walker MJC, Berkelhammer M, Bjork S, Cwynar LC, Fisher CA, Long AJ, et al Formal subdivision of the Holocene Series/Epoch: Discussion paper by a working group of INTIMATE (Ingration of ice-core, marine and terrestrial records and the Subcommission on Quaternary Stratigraphy (International Commission on Stratigraphy). J Quaternary Sci 2012; 27(7): 649–659.

[52] Goriunova OI. Drevnie Mogil’niki Pribaikal’ia. Irkutsk: IGU; 2002. Russian.

[53] Weber AW, Goriunova OI, McKenzie HG, editors. Khuzhir-Nuge XIV, a Middle Holocene Hunter-Gatherer Cemetery on Lake Baikal, Siberia: Archaeological Materials Northern Hunter-Gatherers Research Series 4. Edmonton: CCI Press; 2008.

[54] Weber AW, Schulting RJ, Ramsey CB, Goriunova OI, Bazaliiskii VI. Chronology of Middle Holocene hunter-gatherers in the Cis-Baikal region of Siberia: Corrections based on examination of the freshwater reservoir effect. Quatern Int. In review.

[55] Grousset R. The Empire of the Steppes: a History of Central Asia. New Brunswick: Rutgers University Press; 2005.

[56] Dashibalov BB. Arkheologicheskie Pamiatniki Kurykan i Khori. Ulan-Ude: BNTS SO RAN; 1995. Russian.

[57] Losey RJ, Nomokonova T, White D. Fish and Fishing in Holocene Lake, Baikal, Siberia. J I Coast Archaeol 2012; 7: 126–145.

[58] Amano M, Miyazaki N, Petrov EA. Age determination and growth of Baikal Seals (*Phoca sibirica*). Adv Ecol Res 2000; 31: 449–462.

[59] Nomokonova TIU, Losey RJ, Goriunova OI, Bazaliiskii VI. Obraz nerpy u naseleniia Priba’kal’ia v golotsene (Vostochnaia Sibir’). Archaeol Ethnog Anthropol Eurasia 2014; 3(59): 21–28. Russian.

[60] Goriunova OI, Ovodov ND, Novikov AG. Analiz faunisticheskikh materiialov s mnogosloinogo poseleniia Tyshkine III (oz. Baikal) In: Medvedev GI, editor. Severnaia Evraziia v Antropogene: Chelovek, Paleotekhnologii, Geoekologiia, Etnologiia i Antropologiia, Volume 1 Irkutsk: Ottisk; 2007 p. 168–174. Russian.

[61] Goriunova OI, Svinin VV. Ol’khonskii Raion: Materialy k Svodu Pamiatnikov Istorii i Kul’tury Irkutskoi Oblasti, Part 3 (Materikovyi Uchastok: ot Mysa Ulan do Reki Bol’shaia Bugul’deika). Irkutsk: Arkom; 2000. Russian.

[62] Okladnikov AP. Petroglify Baikala—Pamiatniki Drevnei Kul’tury Narodov Sibiri. Novosibirsk: Nauka; 1974. Russian.

[63] Goriunova OI, Novikov AG, Vorob'eva GA, Weber AW, Losey RJ, Nomokonova TIU, et al Prodolzhenie raskopok Rossiisko-Kanadskoi ekspeditsii v bukhte Sagan-Zaba na Baikale. Problemy Arkheologii, Etnografii, Antropologii Sibiri i Sopredel'nykh Territorii 13; 2007 p. 212–215. Russian.

[64] Goriunova OI, Novikov AG, Vorob'eva GA, Weber AW, Orlova LA. Zavershenie raskopok Rossiisko-Kanadskoi ekspeditsii v bukhte Sagan-Zaba na Baikale. Problemy Arkheologii, Etnografii, Antropologii Sibiri i Sopredel'nykh Territorii 14; 2008 p. 32–35. Russian.

[65] Buck CE, Christen JA, James GN. BCal: an on- line Bayesian radiocarbon calibration tool. Internet Archaeology 1999; 7 (http://intarch.ac.uk/journal/issue7/buck/).

[66] Lyman RL. Quantitative Paleozoology. Cambridge: Cambridge University Press; 2008.

[67] Lyman RL. Vertebrate Taphonomy. Cambridge: Cambridge University Press; 2004.

[68] Lam YM, Pearson OM. Bone density studies and the interpretation of the faunal record. Evol Anthropol 2005; 14: 199–108.

[69] Klevezal’ GA. Registriruiushchie Struktury Mlekopitaiushchikh v Zoologicheskikh Issledovaniiakh. Moscow: Nauka; 1988. Russian.

[70] Scheffer V. Growth layers on the teeth of pinnipeds as an indication of age. Science 1950; 112(2907): 309–311. 14781740 10.1126/science.112.2907.309-a

[71] Storå J. Skeletal development in grey seal Halichoerus grypys, the ringed seal Phoca hispida botnica, the harbour seal Phoca vitulina vitulina, and the harp seal Phoca groendlandica: epiphyseal fusion and life history. Archaeozool 2000; 11: 199–222.

[72] Hodgetts LM. Dorset Palaeoeskimo harp seal exploitation at Phillip’s Graden (Eebi-1), Northwestern Newfoundland In: Monks G, editor. The Exploitation and Cultural Importance of Sea Mammals. Oxford: Oxbow Books; 2005 p. 62–76.

[73] Pastukhov VD. Kraniometricheskaia kharakteristika Baikal’skoi nerpy (Pusa sibirica; Pinnipedia, Mammalia). Zoologicheskii Zhurnal 1969; XLVIII (5): 722–33. Russian.

[74] Goriunova OI, Dolganov VA, Novikov AG, Weber AW. Ranii neolit Priol’khon’ia: po materialam VI kul’turnykh sloev geoarkheologicheskogo ob”ekta Sagan-Zaba II In: Medvedev GI, editor. Evraziia v Kainozoe: Stratigrafiia, Paleoekologiia, Kul’tury Volume 1 Irkutsk: IGU; 2012 p. 86–93. Russian.

[75] Dolganov VA, Goriunova OI, Novikov AG, Weber AW. Punktirno-grebenchataia keramika i ee mesto v neolite Priol’khon’ia (po materialam mnogosloinogo poseleniia Sagan-Zaba II) Vestnik NGU. Seriia: Istoriia, Filologiia 10 (3: Arkheologiia i Etnografiia);v2011 p. 84–91. Russian.

[76] Dolganov VA, Goriunova OI, Novikov AG, Weber AW. Kompleksy s keramikoi posol’skogo tipa v neolite Pribaikal’ia: po materialam V verkhnego sloia geoarkheologicheskogo ob”ekta Sagan-Zaba II) Vestnik NGU. Seriia: Istoriia, Filologiia 12 (7: Arkheologiia i Etnografiia); 2013 p. 125–132. Russian.

[77] Bezrukova EV, Tarasov PE, Solovieva N, Krivonogov SK, Riedel F. Last glacial-interglacial vegetation and environmental dynamics in southern Siberia: chronology, forcing and feedbacks. Palaeogeogr Palaeocl 2010; 296: 185–198.

[78] Bezrukova EV, Hildebrandt S, Letunova PP, Ivanov EV, Orlova LA, Müller S, et al Vegetation dynamics around Lake Baikal since the middle Holocene reconstructed from the pollen and botanical composition analyses of peat sediments: Implications for paleoclimatic and archeological research. Quatern Int 2013; 290–291: 35–45.

[79] Demske D, Heumann G, Granoszewski W, Nita M, Mamakowa K, Tarasov PE, et al Late glacial and Holocene vegetation and regional climate variability evidenced in high-resolution pollen records from Lake Baikal. Global Planet Change 2005; 46: 255–279.

[80] Shichi K, Takahara H, Krivonogov SK, Bezrukova EV, Kashiwaya K, Takehara A, et al Late Pleistocene and Holocene vegetation and climate records from Lake Kotokel, central Baikal region. Quatern Int 2009; 205 (1–2): 98–110.

[81] Tarasov P, Bezrukova E, Karabanov T, Nakagawa E, Wagner M, Kulagina N, et al Vegetation and climate dynamics during the Holocene and Eemian interglacials derived from Lake Baikal pollen records. Palaeogeogr Palaeocl 2007; 252: 440–457.

[82] Tarasov PE, Bezrukova EV, Krivonogov SK. Late Glacial and Holocene changes in vegetation cover and climate in southern Siberia derived from a 15 kyr long pollen record from Lake Kotokel. Clim Past 2009; 5: 285–295.

[83] White D, Bush A. Holocene climate, environmental variability and Neolithic biocultural discontinuity in the Lake Baikal region, Siberia In: Weber AW, Katzenberg MA, Schurr T, editors. Prehistoric Hunter—Gatherers of the Baikal Region, Siberia: Bioarchaeological Studies of Past Lifeways Philadelphia: University of Pennsylvania Press; 2010 p. 1–26.

[84] Devlet E. Rock art and the material culture of Siberian and Central Asian shamanism In: Price NS, editor. The Archaeology of Shamanism. London: Routledge; 2001; p. 43–54.

[85] Mikhailov TM. Buriatskii Shamanizm: Istoriia, Struktura i Sotsial’nye Funktsii. Novosibirsk: Nauka; 1987. Russian.

[86] Nomokonova TIU, Goriunova OI. Kosti zhivotnykh v plitochnykh mogilakh Priol’khon’ia: po materialam pogrebeniia Sarma X In: Konstantinov MV, editor. Drevnie Kul’tury Mongolii i Baikal’skoi Sibiri. Volume 4, part 1 Chita: Izd-vo ZGU; 2013 p. 329–336. Russian.

[87] Mackay AW, Bezrukova EV, Boyle JF, Holmes JA, Panizzo VN, Piotrowska N, et al Multiproxy evidence for abrupt climate change impacts on terrestrial and freshwater ecosystems in the Ol'khon region of Lake Baikal, central Asia. Quatern Int 2013; 290–291: 46–56.

[88] Erlandson JM. The archaeology of aquatic adaptations. J Archaeol Res 2001; 9(4): 287–350.

